# Therapeutical Requisites Necessary to the Salvation of Every Dental Practitioner

**Published:** 1881-02

**Authors:** F. A. Brewer


### THE DENTAL REGISTER.

VOL. XXXV.] FEBRUARY, 1881. [NO. 2.

### THERAPEUTICAL REQUISITES NECESSARY TO THE SALVATION OF EVERY DENTAL PRACTITIONER.

BY F. A. BREWER, D. D. S.

The great mission of therapeutics is in the fulfillment looking to the preservation of the condition of the human system, and whatever we may contribute toward such an object, so much shall it redound to our credit as true philosophers. But then, gentlemen, in order to carry out our mission properly, we must know and acquaint ourselves with the true normality of the system, or any part of it, when in health. By such acquaintanceship, we may be the better able to define any deviation manifesting disordered action. Of course, no practitioner can treat a disease intelligently unless he has become acquainted with its general or special characteristics; but it is not only requisite that he should familiarize himself thus specially, but also to fully observe the defined lines of limitation by which his treatment may influence according to need. But, unfortunately for the progress of our profession, our ranks are filled with many who fail to discriminate that line of demarkation, and their poor patient is forced to stand the brunt of battle. But our profession *is improving.* It would seem customary, with our practitioners, when a certain remedy was recommended for a special purpose, to over-indulge its use; but not only stop there, but make a hobby of it; ride it to death; ignoring entirely as to the effect it might induce by a variation of circumstances, as well as the damages it might inflict in another direction, thus making the remedy worse than the disease itself.

A few months since, a practitioner stated to me that he had seen a statement in one of the journals that sulphuric acid was recommended as a specific for inflammation of the gums, and he believed he would try it. My time being limited, I merely remarked that I should employ it very carefully, as I deemed it very dangerous. A few weeks since, I met the same gentleman, and he informed me, upon inquiry as to his acid remedy, that he had made it a universal remedy in all cases suffering from diseased gums. I inquired if he regulated the strength in any of his cases. He said, no. His formula being one teaspoonful to the ounce. That man, we should say, is preparing for an active practice. A forethought has not entered this man's mind relative to the amount of acid already bathing that gum tissue, or the permanent injury his pet remedy would inflict upon those teeth; nor did he look ahead a little farther to ascertain if a far more efficacious and harmless remedy was not near at hand. *But he liked it*, and his neighbors liked it, too; and he intended to continue to use it.

Stepping in a man's office a few days since, I noticed an operator pour a few drops of creosote into a bottle of tooth wash that he was preparing for one of his patients. Upon inquiry, ascertained it proved the best tooth preserver and deoderizer he knew of. I inquired why he preferred creosote to carbolic acid. He replied that it was an old stand-by, and had been popular; and, furthermore, he was of the opinion that it possessed better virtues for destroying any fungus that might collect upon the interstices of the teeth. In fact, said he, I find it a balm remedy for the prevention of any disease affecting the oral cavity. I remarked that perhaps he had better try it on the stomach, also. In a conversation with another gentleman a short time since, I was told that a better boon was never bestowed upon the dental profession than the introduction of carbolic acid. He had found it a sovereign remedy for prevention of decay in and around a filling after its insertion, as well as also a superior remedy for the prevention of scurvy of the teeth. I inquired of him if he did not think the scalpel and scaler would prove a better substitute? Oh, he replied, those dangerous instruments may be dispensed with, as they are not needed. Pressing my inquiry farther as to how his remedy, carbolic acid, prevented decay around his filling, he replied, decay is the result of a fungus, and by swabbing out the cavity well before the insertion, fungus will not enter there; they would become disgusted. I replied, I thought his theory a good one as to the fungus, *provided* he could control two disadvantages that would necessarily ensue, which was, the retention of the strength of his medicine, as well as the doorway encircling his filling by the presence of his remedy. Oh, said he, I should not employ it crystalized. In fact, said he, it has become the true friend of my office. I replied I feared it was like many other remedies in our list of *materia medica*, dangerous precedents in careless hands.

Thus we perceive the necessity of discreet men in our profession. Men who have the brains to reach farther on in the scale of selection as well as experimentation, and who will not become so attached to any one remedy because it seems to fulfill a certain requirement.

While referring to carbolic acid, gentlemen, allow me to digress by saying that this has become the darling remedy for every complaint known to oral science or dentology. If the gum tissue be but slightly congested, this crystalized diamond must shed its rays over and beneath its surface. If Odontalgia manifests itself within the denture, no matter if the primary cause arise far remote from the locality of manifestation, carbolic must come to the rescue where the pain is noticed. If a nerve takes a notion to thrust its little head out of its locker, and take an innocent peep at a passing stranger, it must be treated to a submarine bath of this favorite liquid, and forced to take a back track at double quick. If, in the excavation of a crown cavity, some of the guardian sentinels of sensation manifest their dignity, their mouths must be stuffed with it as a pickle, to quiet them in their little bed. If a tonic is needed, carbolic acid is the antidote wanted. If an astringent, this special balm will fill the bill. If a tooth wash is called for, a mite of carbolic acid gives it *tone*. Ask its affiliator *why* he uses it, and, nine times out of ten, he will answer because it is *popular*. So magnetic is its influence, so devoted its friendship, that it will not only remain with us at our office, but persists strongly in following us to our club room, as well as parlor, and to the social party of our kind neighbor. Truly, is not carbolic acid *king* of all it surveys? Although it may possess many virtues as an antiseptic or disinfectant; though it may possess strength of power not found in other medicines of its character; yet there are times it should not be employed at all, as by its employment it may prove the very agency of thwarting the desired result that we seek for. Hence, I say, when picking up the bottle, stop and think whether you really need it at all, and whether there are not other medicines which might be more carelessly used, and yet prove *harmless*.

If we perceive some one of our profession employing iodine for any one special purpose, resting his belief that it is good as a remedy, iodine is at once adopted for all such purposes regardless of varying circumstances. Now, gentlemen, such variation may prove one of the greatest reasons *why* it should not be employed at all! If chloride of zinc be recommended — not only as an obtunder of sensitive dentine, but for ulceration of gingivitis — it becomes at once the pet remedy under all circumstances, whether the true primary cause of the origination had been removed or not, as also for every condition of inflammation. As for illustration: a lady patient finding the marginal gingival tissue surrounding a canine tooth to have become more than ordinarily inflamed in a day or two following the insertion of a filling, the disturbance having arisen probably through carelessness in applying the rubber dam, or from tartar having been driven in contact with the periosteal membrane, returned to her dentist to be relieved of her constantly increasing pain; forthwith a solution of chloride of zinc was applied to the inflamed part. As a result, the pain not only increased by additional inflammation, but an undue shrinkage of the tissue by the dissolution of the periphery of the alveola followed. *Why* he did not put a little carbolic acid in it, I am not prepared to say. A short time since a lady was suffering from slight accumulations of tartar causing her much trouble with some of her teeth. Here again, a diluted wash of chloride of zinc was given her in such strength that pain followed every application of it. I presume his idea was to destroy the tartar. I cannot conceive of any other purpose, unless he had so habituated himself to its use, that he could think of no other remedy, or he may, like many others, have tried to carry his point by experimenting. A few strokes of a scaler would in this instance have eased the suffering patient, if not depletion, or the application of an anodyne might have had the desired effect. This done, the dentist immediately proceeds to filling the teeth, regardless of their surrounding conditions, failing to observe whether bathed in a constant flood of acid, or resting upon a rotten foundation induced through a strumous diathesis, or influenced by a highly nervous temperament, or that of a low febrile tendency, all the while indifferent whether that system has been strained to its utmost by some mental tax, and thus caused his patient to shrink from the very touch or even sight of an excavator. Yet, notwithstanding these unfavorable conditions, we too frequently, through our rules of office law, go through our manipulations, upon the case mentioned, with a guess *that'll do*, as Atkinson says. Yet when the question in all its sacredness comes to us in due shape, as to whether we truly fulfilled what the patient anticipated, as well as sustained our honor and reputation, are we able to say, Yes? Should we not rather have temporarily prepared those teeth, awaited another day, when we would have found our case in a better condition for the fruits of our operations to prove permanently successful. Gentlemen, through the ignoring of these special conditions as well as our hasty and indifferent manipulations, may it not be attributable to the rising of that *creature* now gaining rapid headway in the East, "*The New Departure?*" When we are so openly and plainly informed, that out of every two thousand cases of our operative manipulators seventy-seven per cent. are failures, does it not seem a bitter pill to swallow? If dental progression appears so unfavorable as claimed that so much time should be devoted to discussions upon the extremes of practice, does it not seem more unfavorable for us to be compelled to shoulder the charge that for upwards of fifty years the profession have been as "the *blind leading the blind?*" *How* I would ask, may we require the right from wrong! unless we resort to *extremes*, and thus sift out the dross.

Science to-day, has become a success, mainly through *practical effort and observation.* But if those special laws were so frequently and carelessly overridden as occurs in our profession to-day, we should *yet* be found with the meal at one end of the sack and the stone at the other. But our most able workers are at last awakening to their duty! It is said, gentlemen, that this is a plodding age, and soon there will appear a mineral balm, substituting the metal basis which we have for so long worked arduously and skillfully to form, an enduring monument of fame! It is hardly necessary to mention here the theoretical fancies urging this new creature onward, as they must be familiar to each one of us. But are we ready for it? Shall we lose that ambition for which we have so long been urging, and which, if this substitution once gets hold of us, must deteriorate? But should it not rather induce a serious reflection on our minds relative to the conditions which have led this check to our boasted progression? Let me ask if there has not been a little too much of that electro element in our manipulations rather than in the material employed? Have we been correct when allowing those two molars to reassume the same closely approximatiug position held previous to the temporary wedging through which we were enabled to insert a deep seated cervical filling, and in their normal position so as a thread could with force but barely pass at their union, and that in a mouth whose test by litmus exhibits a redundancy of acid? Have we found ourselves justified in allowing those distal borders at the grinding edge of those bicuspids forming a thin flooring for sustaining approximal fillings to remain, which in a few months, or even years, disappear through natural forces? Or were we not in error when, through an arbitrary restrictiveness to one kind of metal basis, we attempted to insert a simple filling at the posterior approximating cervical region, and by doing so, and for want of the proper get-at-able as well as the needs of another class of material, we might have prevented not only a leak, but an eventual nerve exposure? All these have their bearing, which in their aggregation form a far more damaging element than the electro. And, gentlemen, to sum up as a finale, we need more space; we need a self-cleansing repelling form to the face of our filling.

A filling of gold inserted in one of my teeth, also two of amalgam, over thirty years since, by the late lamented Chapin A. Harris, are still doing duty; and the electrical influence is still so great in the gold filling, that the close approximation of a tack to its surface presents quite an unpleasant sensation, but nevertheless remains perfect. But one valuable lesson have I learned from their presence and attending effects, and that is the necessity that exists for space and voluntary power of self-cleansing.

In conclusion, let me say, gentlemen, go to work to-morrow determined to extend our investigations far in advance of the past, as well as using wise discretion in all of our manipulations, as well as moderation in our machinal applications, so that we may advance the standard of our claims, and thus thwart the opposing elements which bear down upon our noble ship, threatening to sink her.--*Cal. State Dental Association.*

